# A Practical Treatise on the Most Common Diseases of the South, Exhibiting Their Peculiar Nature, and the Corresponding Adaptation of Treatment

**Published:** 1851-05

**Authors:** 


### REVIEWS.

---

ART. IV.—*A Practical Treatise on the most common Diseases of the South,* exhibiting their peculiar natnre, and the corresponding adaptation of Treatment. To which is added an Appendix, containing some miscellaneous matter. Also a Glossary explaining the meaning of the Technicalities, or medical phrases, used in this book. By THOMPSON MCGOWN, M. D., Graduate of Transylvania University, Member of the Lexington Medical Society, and a Practitioner of the South. "Is there no *balm* in Gilead? is there no *physician* there?" Why, then, may not all be healed, or profited? Philadelphia: Grigg, Elliot, & Co. 1849. 8 vo. p.p. 441.

This, it must be confessed, is not a very prepossessing titlepage. The author has undertaken one of the most difficult of tasks—to write a book on medicine at once scientific and popular. Horace considered that an author had attained every point who had succeeded in mingling the useful with the agreeable. It was deemed by the ancients one of the most dangerous feats in navigation to sail between Scylla and Charybdis. No doubt both these achievements demand great talent, skill and address, but we question whether either is as difficult as it is to compose a scientific work which, while it is adapted to the capacities of the populace, is also fitted to instruct the professional reader. Dr. McGown thinks he has succeeded in both particulars, and that his book "will be of great value to students of medicine, young, and even old practitioners, and those who have recently emigrated to the South or south-west, and the southern and southwestern people generally." We are not going to be so illnatured as to gainsay this, or in any way attempt to disturb the feeling of complacency with which our brother regards the offspring of his pen. We hope his treatise will prove to be all that he believed respecting it when he sent it abroad upon its benevolent mission.

Dr. McGown is a decided believer in sectional medicine. He holds that medical books to be valuable in the South must be written in the South, and ascribes to Dr. Cartwright the sentiment that "the best works extant on *diseases of the South*, are those of Hippocrates, the father of medicine." We have more than once expressed our emphatic dissent to this doctrine, and we can imagine the feeling of surprise with which the eminent physician just alluded to will find himself quoted for such an opinion. Dr. Cartwright has declared that Hippocrates has given the "best descriptions" of southern diseases, but to say that "his works on diseases of the South are the best extant," would be a great libel on the profession. It would indeed be time to "throw physic to the dogs," if no improvement had been made in the treatment of "diseases of the South" during the long centuries of professional toil and study that have elapsed since the days of the Coan sage. And medicine, we think, must resign its high pretensions to science if it can be shown, that it is a mere local, sectional thing, destitute of principles of general application, which must be studied in special localities, under teachers educated with special reference to particular diseases—if it can be shown, that only northern physicians are qualified to teach and practice their profession in the north, and none but physicians of the south are fit for southern practice and teaching. Still, a treatise on the particular diseases of the South written by a competent observer who has had long experience in their management, must possess its advantages. It is likely to be more graphic and truthful than the production of a writer who had never witnessed the phenomena he describes; and hence every contribution to our medical literature from the pens of southern physicians is welcomed by the profession in every quarter of the country.

The principal subjects treated of in this volume are the following:—Malarious diseases, Intermittent Fever, Cachexia, Tuburculous Cachexia, Scrofula, Chronic Rheumatism, Tabes Mesenterica, Chronic Bronchitis, Hemoptysis, Dropsy, Enlargement of the Spleen and Liver, Epilepsy, Puerperal Convulsions, Dyspepsia, Leucorrhea, Dysmenorrhea, Congestive Fever, Remittent Fever, Yellow Fever, Acute Rheumatism, Typhoid Pneumonia, Diarrhea, Dysentery, Asiatic Cholera and Croup. In an appendix some notice is taken of certain miscellaneous matters, as Gonorrhea, Concussion, Incised Wounds, Gun Cotton, Collodion, Worms, Catarrh, Ephemeral Fever, and Itch. A glossary is added for the benefit of non-professional readers.

If it was thought worth while, we might institute an inquiry as to how far some of the above recited affections can properly be claimed as "diseases of the South." In what sense, for example, is chronic rheumatism, or dyspepsia, or typhoid pneumonia, or epilepsy, or dysmenorrhea, or itch, a Southern disease? Doubtless they all occur in the South, as they occur in all countries, but there is no propriety in bringing them into such a category except for the purpose of pointing out the peculiarities imparted to them by the climate and other influences of the South, and this our author has hardly essayed to do. We expect a treatise on diseases of the South by a southern physician to contain something distinctive. We expect to find in it something better than vague generalities, or even sound opinions and good extracts gathered from English, French, and American authors. It ought to be an exponent of the professional opinion and practice of the South. It ought to consist of graphic descriptions of the complaints of the country, their peculiarities, the treatment applicable to their modifications, their statistics, and, as far as dissections have revealed anything, the post-mortem appearances. In a word, it ought to be the record of an observer on the spot, and to contain what the eye-witness alone can impart. Such a work is highly interesting and instructive. It is sought after with avidity, because it comprises new facts and faithful histories—is a mine of original observations. The author of such a work must be a man who can "employ sometimes the eye of the body, and at others that of the mind," who has a talent for detail as well as powers of observation, and "who unites with the industry of the mechanic the soul of the philosopher."

Our conviction is that the treatise before us cannot be ranked very high among the works which "the diseases of the South" have called forth. It appears to us as evincing a want of judgment, or tact, or, to say the least, of self-reliance, that the author deemed it necessary to introduce his work with certificates as to his professional standing from "ministers of the gospel," "sheriffs," "ex-clerks of courts," and "attorneys at law." This may do very well for the popular wing of his readers, but we fear that it will have any other effect than to advance his work with his professional brethren.

